# Diabetic Vitrectomy is Over and the Retina Remains Attached; What is Next?

**Published:** 2010-04

**Authors:** Mehdi Modarres-Zadeh

**Affiliations:** Eye Research Center, Rassoul Akram Hospital, Iran University of Medical Sciences, Tehran, Iran

At the happy ending of vitreoretinal surgery for proliferative diabetic retinopathy, when all proliferative fibrovascular tissues have been successfully removed, panretinal photocoagulation is completed and there is no iatrogenic break, the surgeon may wonder whether to consider any other intervention before closing the case. This question arises in microincision vitrectomies as well as with traditional 20 gauge techniques. Several maneuvers have been proposed in the literature including gas-fluid exchange with a non-expandable air-gas mixture, cryotherapy of the sclerotomy sites, injection of bevacizumab and/or triamcinolone acetonide, or any combination thereof. Microincision vitreoretinal surgery may be associated with a higher incidence of postoperative hypotony; 1 therefore many surgeons prefer to perform gas-fluid exchange at the end of surgery. In 20 gauge vitrectomy, the incisions are secured with sutures and the chance of hypotony is low. There is no general agreement on the necessity of or any benefit from gas-fluid exchange.

In this issue of the Journal of Ophthalmic and Vision Research, Farrahi and associates^2^ have compared the incidence of postoperative vitreous hemorrhage between two groups of patients, 50 each, with and without gas-fluid exchange at the end of diabetic vitrectomy. They found that vitreous hemorrhage occurred less frequently in eyes with gas-fluid exchange as compared to eyes in which the vitreous cavity was left filled with lactated Ringer’s solution. The difference was statistically significant on the 4th and 7th postoperative days but not 4 weeks after the operation. However, the major limitation to their study is not grading the severity of fibrovascular proliferation, and lack of randomization of eyes on that basis. Still, it is likely that gas-fluid exchange at the conclusion of vitrectomy may actually decrease the chance of postoperative hemorrhage during the early postoperative period. As already mentioned, currently there is no strong evidence for or against this maneuver.

Other interventions have also been studied. Cryotherapy of the sclerotomy sites for prevention of late postoperative vitreous hemorrhage after diabetic vitrectomy was tried by Entezari and coworkers^3^ yielding no benefit. On the other hand, injection of bevacizumab and triamcinolone at the end of diabetic vitrectomy was found to reduce the incidence of postoperative vitreous hemorrhage by Park et al.^4^ Also in a study by Faghihi et al,^5^ intravitreal injection of triamcinolone acetonide at the conclusion of surgery decreased the rate of postoperative hemorrhage. However, Romano and colleagues^6^ did not observe such an effect from injection of bevacizumab at the end of surgery.

There is no strong evidence indicating the necessity of any of these procedures at the end of diabetic vitrectomy. On a biological basis one would consider the injection of bevacizumab and/or triamcinolone acetonide to be useful adjuncts to surgery. Fluid-gas exchange also seems to be advisable in microincision vitrectomies. Other procedures depend on the individual surgeons’ discretion. Well-powered randomized clinical trials exploring the benefit of such adjunctive procedures are direly awaited in the ophthalmic community.
